# Ecological and dynamic analysis of gut microbiota in the early stage of azomethane-dextran sodium sulfate model in mice

**DOI:** 10.3389/fcimb.2023.1178714

**Published:** 2023-04-20

**Authors:** Ruizheng Sun, Hao Chen, Siqi Yao, Zheng Yu, Chen Lai, Jing Huang

**Affiliations:** ^1^ Department of General Surgery, Xiangya Hospital, Central South University, Changsha, Hunan, China; ^2^ Department of Parasitology, School of Basic Medical Science, Central South University, Changsha, Hunan, China; ^3^ Hunan Key Laboratory of Precise Diagnosis and Treatment of Gastrointestinal Tumor, Xiangya Hospital Central South University, Changsha, Hunan, China; ^4^ International Joint Research Center of Minimally Invasive Endoscopic Technology Equipment & Standardization, Changsha, Hunan, China

**Keywords:** gut microbiota, dynamic analysis, azomethane, dextran sodium sulfate, mice

## Abstract

The success rate of azomethane-dextran sodium sulfate (AOM-DSS) model in mice has been a long-standing problem. Treatment of AOM and the first round DSS induces acute colitis and is of great significance for the success of AOM-DSS model. In this study, we focused on the role of gut microbiota in the early stage of AOM-DSS model. Few mice with obvious weight loss and high disease-activity score survived from double strike of AOM and the first round DSS. Different ecological dynamics of gut microbiota were observed in AOM-DSS treated mice. *Pseudescherichia*, *Turicibacter*, and *Clostridium_XVIII* were of significance in the model, uncontrolled proliferation of which accompanied with rapid deterioration and death of mice. *Akkermansia* and *Ruthenibacterium* were significantly enriched in the alive AOM-DSS treated mice. Decrease of *Ligilactobacillus*, *Lactobacillus*, and *Limosilactobacillus* were observed in AOM-DSS model, but significant drop of these genera could be lethal. *Millionella* was the only hub genus of gut microbiota network in dead mice, which indicated dysbiosis of the intestinal flora and fragility of microbial network. Our results will provide a better understanding for the role of gut microbiota in the early stage of AOM-DSS model and help improve the success rate of model construction.

## Introduction

Colorectal cancer (CRC) is the third most common malignancy and the second leading cause of cancer deaths worldwide (Sung et al., 2021). Taking tumor heterogeneity into consideration, experimental animal models of CRC are still main ways to study the pathogenesis of CRC. Azomethane (AOM) combined with dextran sodium sulfate (DSS) in rodents is one of the most common models to construct chemical-induced colorectal neoplasm. The model established by AOM-DSS has been widely used for pathological and genetic studies of CRC (Chung et al., 2021; Gong et al., 2022). Methyl diazo ions are generated following AOM treatment, which lead to aberrant DNA methylation and ultimately initiate and promote the development of CRC (Fiala et al., 1987; Reddy, 2004). Abnormal expression of β-linked proteins caused by DSS induces local inflammatory environment and exacerbates the process of tumorigenesis (Reddy, 2004). However, it’s difficult to establish this model due to the dual use of AOM and DSS and the variable sensitivity of mice from different genetic and environmental backgrounds (Amos-Landgraf et al., 2014; Neufert et al., 2021). It has been documented that the development of moderate colitis level during each DSS cycle is crucial in AOM-DSS model (Neufert et al., 2021). Specifically, occurrence of DSS-induced acute colitis in the pre-construction phase of AOM-DSS model is of great significance to successful colorectal tumorigenesis.

Increasingly number of studies have shown that the gut microbiota plays an important role in CRC induced by AOM-DSS. The disturbance of the gut microbiota is one of the important reasons for the deterioration of CRC. Decrease in beneficial bacteria (anaerobic species) and increase in parthenogenic anaerobes (e.g., pathogenic *Enterobacteriaceae*) are main characteristics of dysbiosis in gut microbiota (Lupp et al., 2007; Zitvogel et al., 2018). It has been shown that pathogenic *Parasutterella* was significantly enriched after AOM-DSS treatment (Ibrahim et al., 2019). This may lead to disruption of intestinal barrier, destruction of the mucus layer and epithelium, and induction of an immune response that may lead to chronic inflammation (Davenport et al., 2014; Shahanavaj et al., 2015). Genera of the *Lachnospiraceae* family, which had been shown to have potential probiotic properties, were found to be significantly reduced after AOM-DSS interference (Ibrahim et al., 2019). However, most of the previous studies reported there were microbial compositional changes after the model was successfully constructed, few studies focused on the constructing process, especially on the early stage (Zackular et al., 2013; Ibrahim et al., 2019). In the constructing process, the barrier function of the intestine and destabilization of the mucus layer in the intestinal wall were significant impairment caused by the intervention of DSS in mice, which made the bacteria more permeable (Morgan et al., 2013; Johansson et al., 2014). The double strike of AOM-DSS increased the mortality of mice in the early stage and was one of the main reasons for the failure of model construction. It is intriguing and important to explore the role of gut microbiota in the early stage of the AOM-DSS model. Therefore, we focused on the dynamic evolution of gut microbiota in this study. Our findings will provide a better understanding of the rapid transformation of gut microbiota in the early stage of AOM-DSS model construction and help build foundation for improving success rate of model construction.

## Methods

### Animal experiments

Male C57BL/6 mice (6 weeks old) were purchased from Hunan Sleek Jingda (SLAC), Changsha, China. All mice were housed in plastic cages with stainless steel grids. The environmental conditions were sterile, with free access to standard rat food and drinking water under controlled temperature (25 ± 5°C), humidity (60%-70%), and light (12/12hour light/dark cycle). Mice were given 7 days to acclimatize to the environment prior to the start of the experiment. The study was approved by the Laboratory Animal Ethics Committee of Xiangya Hospital, Central South University (No. 2022060872). All animal experimental operations were performed in accordance with the Institutional Guidelines for the Care and Use of Laboratory Animals.

At the end of the acclimation period, mice were randomly divided into Control group and DSS group. The DSS group was then divided into DSS_Alive group and DSS_Dead group according to the survival status of the first round DSS. The Control group continued having free access to standard rodent chow and sterile drinking water. Mice in the DSS group were fed 2% DSS drinking water daily for one week after intraperitoneal injection of the mutagen AOM (10 mg/kg). Then they consumed sterilized drinking water for two more weeks. AOM was purchased from Sigma-Aldrich (St. Louis, MO) and DSS was from MP Biomedicals (Santa Ana, CA).

### Sample collection and physical measurement

Fecal samples were collected every two days, and feces were stored at -80°C for further analysis. Body weight, the presence of blood in the stool, and stool consistency were measured every two days. Disease activity index (DAI) was then evaluated based on the three parameters, similar to the subjective clinical signs observed in human ulcerative colitis (Howarth et al., 2000).

### PCR and high-throughput sequencing of 16S rRNA

Microbial DNA extraction was performed using HiPure Stool DNA Extraction Kit (Magen, Guangzhou, China). The V3-V4 region of the ribosomal RNA gene were amplified by polymerase chain reaction (PCR, 95°C for 5 min, followed by 30 cycles at 95°C for 1 min, 60°C for 1 min, and 72°C for 1 min and a final extension at 72°C). PCR was conducted using the forward primers 341F (5’-CCTACGGGNGGCWGCAG-3’) and reverse primers 806R (5’- GGACTACHVGGGTATCTAAT-3’). Related PCR reagents were from New England Biolabs, USA. Amplicons were collected from 2% agarose gels, and purified by the AxyPrep DNA Gel Extraction Kit (Axygen Biosciences, Union City, CA, USA) according to the manufacturer’s instructions and quantified using ABI StepOnePlus Real-Time PCR System (Life Technologies, Foster City, USA). The purified libraries were then pooled in equimolar and paired-end sequenced on the Illumina MiSeq system using the PE250 sequencing strategy (MiSeq Reagent Kit) by Guangzhou Kidio Technology Services Co. After sequencing, the data were decomposed into appropriate samples based on barcodes and the appropriate sequences were imported into downstream software. To get high quality clean reads, raw reads were further filtered according to the following rules using FASTP (version 0.18.0) (Chen et al., 2018). Quality control and denoise of raw reads were performed based on standard amplicon pipeline as previously described (Liu et al., 2021). Specifically, the denoising method was *-unoise3* available in USEARCH (Edgar and Flyvbjerg, 2015). The representative ASV sequences were classified into organisms by a naïve Bayesian model using RDP classifier (version 2.2) based on SILVA database (version 138.1) (Pruesse et al., 2007; Wang et al., 2007). The feature table and taxonomy annotation table were used for further data analysis.

### Data analysis

All statistical analyses were performed using the R V4.1.2 environment (R Core Team, 2021). The statistical results were visualized using the “ggplot2” package unless specified otherwise. Rarefication, Shannon Wiener index, and beta diversity based on Bray-Curtis distance were generated using package “Vegan”. The package “randomForest” was used for random forest regression analysis, and the package “Pheatmap” was used for visualization of genus biomarkers. Functional analysis of microbial community was conducted based on ImageGP platform integrating several predictive algorithms including functional annotation of prokaryotic taxa (FAPROTAX), phylogenetic reconstruction of unobserved states (PICRUST), and BugBase (Langille et al., 2013; Louca et al., 2016; Ward et al., 2017; Chen et al., 2022).

The co-occurrence networks were established based on Spearman correlation analysis by the “igraph” package. Genera were screened prior to analysis, and only genera with relative abundances above 0.005 were retained. The Benjamin and Hochberg false discovery rate (FDR) was calculated to correct the P-value of Spearman analysis; Cutoff of correlation coefficient and corrected P-value was 0.6 and 0.05, respectively. Network topology properties and hub networks were generated with Gephi software (version 0.10.0).

## Results

### Effect of AOM-DSS modeling on physiological indications

**Figure 1:**
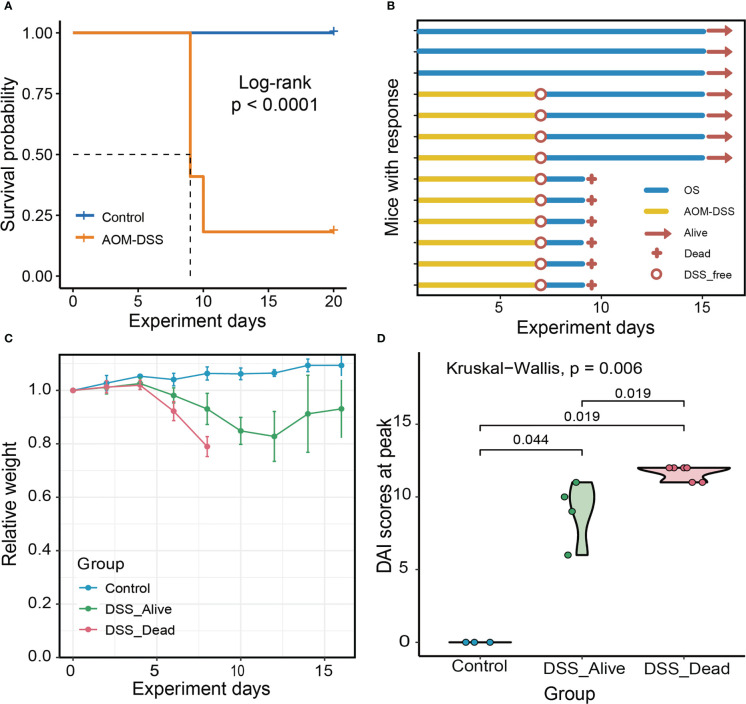
Experiment overview and mice characteristics of the three group. **(A)** Survival plot of AOM-DSS treated mice and control. **(B)** Experimental protocols, progress and results for Control group, DSS_Alive group and DSS_Dead group. **(C)** Changes in body weight of the three group as the experiment was conducted over time. **(D)** Violin plot of the highest DAI score of the three group. Kruskal-Wallis test was used to tested the differences of the DAI scores at peak in three groups.

In this study, we used AOM-DSS to construct an experimental model of CRC in mice. AOM-DSS treated mice had significantly poor prognosis, and the median overall survival (OS) time of which was eight days (**Figure 1A**). After receiving AOM injection, a large number of mice showed significant weight loss and appeared to die with the use of DSS (**Figure 1B**). The combined application of AOM and DSS in the early stage might be the key for CRC modeling. Throughout the experiment, we found that there were obviously different trends in the body weight changes of the three groups (**Figure 1C**). The body weight of mice in the Control group basically remained stable and showed a slightly increasing trend. Body weight of DSS group began to decline after receiving DSS drinking water. The disease activity index (DAI) of the mice was measured and there were significant differences among the three groups. The highest DAI score fluctuated from six to ten in the DSS_Alive group, and around eleven in the DSS_Dead group (**Figure 1D**). Collectively, the combination of AOM with DSS ultimately contributed to high mortality rate in the pre-modeling period of CRC. And the death of DSS-treated mice might be correlated with more serious colitis.

### The diversity and composition dynamics of gut microbiota

#### The diversity of gut microbiota

**Figure 2:**
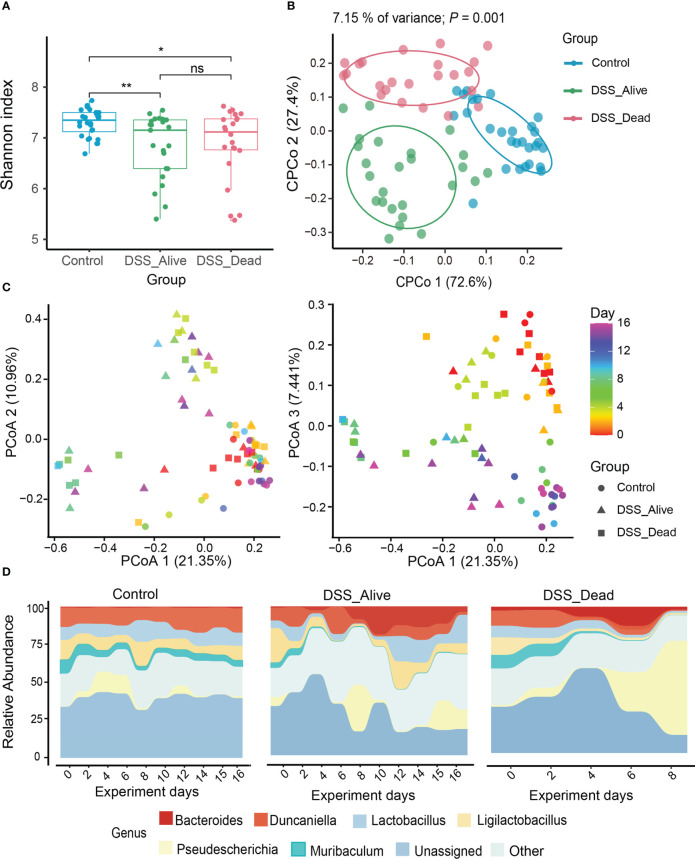
Diversity and composition dynamics of the three group. **(A)** The comparison of Shannon index among three groups. **(B)** The analysis of CPCoA between three groups. **(C)** Beta diversity of three groups analyzed by PCoA. Color bars represent experimental days, and different groups are represented by different symbols. **(D)** Bacterial community composition at the genus level among Control group, DSS_Alive group and DSS_Dead group. Wilcoxon rank-sum test was used to test significance in boxplot. n.s. means no significance, * means P < 0.05, ** means P < 0.01.

Rarefaction curves revealed that sequencing depth of the three group was adequate (**Figure S1**). Rarefied feature table was generated in order to avoid bias of different sequencing depth. In the analysis of microbial diversity, alpha diversity was illustrated by the Shannon Wiener index. Alpha diversity between the Control group and the other two groups were of significant difference (**Figure 2A**). The Shannon Wiener index increased slowly over time in the Control group. However, the Shannon Wiener index decreased in the DSS_Alive group after receiving DSS and finally remained flat, while the DSS_Dead group decreased sharply after the fifth day of the experiment (**Figure S2A**). To evaluate the structural similarity of intestinal microbial communities among the Control group, DSS_Alive group, and DSS_Dead group, principal coordinate analysis (PCoA) was performed based on Bray-Curtis distance. In the constrained PCoA (CPCoA) analysis, we can see that the Control group, DSS_Alive group, and DSS_Dead group were completely separated (**Figure 2B**). There were significant differences in the gut microbial composition among the three groups (**Table S1**). However, microbial compositions of the AOM-DSS treated mice in different cages were similar (**Figure S3**, **Table S1**). The Bray-Curtis distance of the three groups increased with the development of the experiment (**Figure S2B**). The PCoA results showed that the samples of Control group clustered together with the progress of the experiment. Specifically, the samples of DSS_Alive group and DSS_Dead group gradually separated from the Control group after the use of DSS in the second coordinate axis, indicating that the induction of AOM-DSS was the key reason for samples shift. Additionally, the samples of DSS_Alive group and DSS_Dead group also shifted with the progress of the experimental time in the third axis, indicating that the intervention time was another important factor for the changes in these two groups (**Figure 2C**).

#### The composition dynamics of gut microbiota

To further confirm the correlation between the gut microbial structure and experimental time among different groups, the Pearson correlation analysis showed that the microbial structure did not change significantly with the progress of experimental time in the Control group. The correlation in the DSS_Alive group and DSS_Dead group remained the same in the early period, but they had different trends in the late of experiment. The Pearson coefficients of DSS_Alive group increased gradually, while the DSS_Dead group decreased continuously (**Figure S4**). It revealed the rapid transformation of microbial structure during the acute phase of colitis. For microbial composition, Bacteroidetes consisted of nearly half of the Control group, while its percentage was less than 40% in the DSS_Alive and DSS_Dead group at the phylum level. The proportions of Proteobacteria was over 20% in AOM-DSS treated group, which was less than 20% in the Control. Compared with the Control group, the relative abundance of Verrucomicrobiota in the DSS_Alive and DSS_Dead group reached nearly 4% (**Figure S5A**). At the genus level, compared with the Control group, the relative abundance of *Duncaniella*, *Ligilactobacillus*, and *Lactobacillus* decreased in DSS_Alive group and DSS_Dead group, but *Pseudescherichia*, *Bacteroides*, and *Akkermansia* increased (**Figure S5B**). In addition, the relative abundance of most genera remained constant in the Control group showed by microbial composition over time, but *Pseudescherichia* mainly appeared in the early period. The relative abundance of *Muribaculaceae*, *Ligilactobacillus*, *Lactobacillus*, and *Pseudescherichia* showed a large variation in the DSS_Alive and DSS_Dead groups. In the DSS_Alive group, the relative abundance of *Ligilactobacillus*, *Lactobacillus*, and *Pseudescherichia* first decreased and then increased. From the fourth day of the experiment, the relative abundance of *Bacteroides* increased substantially, while *Duncaniella* slowly declined. However, in the DSS_Dead group, we could see that the relative abundance of *Ligilactobacillus*, *Lactobacillus*, and *Muribaculaceae* decreased, while *Pseudescherichia* and *Bacteroides* began to increase on the fourth day of the experiment (**Figure 2D**). The relative abundance of *Bacteroides* was elevated in the DSS_Alive and DSS_Dead groups in the later period of the experiment, which was rare in the Control group.

### Gut microbiota with significant differences in abundance

**Figure 3:**
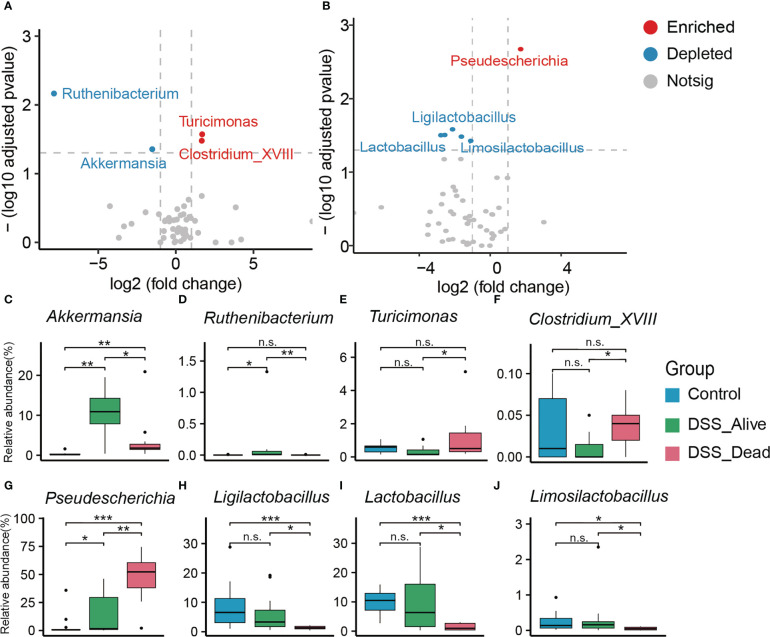
Significant gut microbiota with differences in relative abundance. Volcano plot between the DSS_Dead group and the DSS_Alive group in the stage of breakout **(A)** and convalescence **(B)**. Boxplot plot of corresponding significant bacteria among the three group **(C–J)**. Wilcoxon rank-sum test was used to test significance in three groups. n.s. means no significance, * means P < 0.05, ** means P < 0.01, *** means P < 0.001.

To specify which microbiota might have the ability to decide fate of AOM-DSS treated mice, we performed differential analysis between the DSS_Alive group and the DSS_Dead group in stages of breakout and convalescence. Compared with the DSS_Alive group in stage of breakout, we found that *Turicimonas* and *Clostridium_XVIII* were significantly enriched while *Ruthenibacterium* and *Akkermansia* were significantly depleted in the DSS_Dead group (**Figure 3A**). As for convalescence stage, *Ligilactobacillus*, *Limosilactobacillus*, and *Lactobacillus* were significantly depleted and *Pseudescherichia* was significantly enriched (**Figure 3B**). The relative abundance of corresponding bacteria was specifically revealed. *Akkermansia* and *Ruthenibacterium* were significantly enriched in the DSS_Alive group (**Figures 3C, D**). The relative abundance of *Akkermansia* was significantly higher in AOM-DSS treated mice. While elevating to more than 10% in the DSS_Alive group, the median abundance of *Akkermansia* was less than 5% in the DSS_Dead group (**Figure 3C**). *Ruthenibacterium* was only significantly enriched in the DSS_Alive group, and there was no difference between the control group and the DSS_Dead group (**Figure 3D**). *Pseudescherichia, Turicimonas*, and *Clostridium_XVIII* were significantly enriched in the DSS_Dead group (**Figures 3E–G**). The significant difference of *Turicimonas* and *Clostridium_XVIII* only appeared between the DSS_Alive group and the DSS_Dead group (**Figures 3E, F**). Application of AOM-DSS induced significant increase of *Pseudescherichia*, and the median relative abundance of *Pseudescherichia* in the DSS_Dead group was over 50% (**Figure 3G**). The relative abundance of *Ligilactobacillus*, *Lactobacillus*, and *Limosilactobacillus* in the DSS_Alive group showed no significant decline when compared with the Control group, while those bacteria in the DSS_Dead group were significantly depleted (**Figures 3H–J**).

### Models about microbial biomarkers of experimental time and DAI scores

To build a model that correlates gut microbiota composition with the progression of CRC, we regressed the relative abundance of gut microbiota in three groups on the time of experiment by using the Random Forests machine learning algorithm. First, we assessed the importance of bacterial classes by cross-validation. We found that the cross-validation error was relatively low when 13 genera were used in the three groups. Therefore, we used these 13 genera as biomarker taxa. Most of the biomarker taxa of the three groups showed high relative abundance in corresponding experimental time. There were both similarities and differences in these time-specific genera of three groups. In the Control group, we found that *Muribaclum*, *Peribacillus*, and *Lysinibacillus* showed high abundance at the beginning (**Figure S6A**). While, *Muribaclum, Prevotellamassili*, and *Limosilactobacillus* showed high abundance in the DSS_Alive group (**Figure S6B**). As the disease worsened, *Clostridium_sensu_stricto*, *Pseudescherichia*, and *Turicibacter* increased rapidly in AOM-DSS treated mice, accompanied by some deaths (**Figures S6B, C**). While mice eventually survived, *Mediterraneibacter*, *Mucispirillum*, and *Weeksella* presented high abundance in the end (**Figures S7A, B**). *Limosilactobacillus* was time-specific genus in the DSS_Alive group and DSS_Dead group, indicating its importance and potential function of maintaining gut homeostasis in AOM-DSS treated mice (**Figures S6B, C**).

We also performed a Random Forest machine learning algorithm based on DAI scores. We assessed the importance of bacterial classes by cross-validation to discover key microorganisms, and the number of classes in the cross-validation error curve was relatively stable (**Figure S7B**). Therefore, we used these 13 genera as biomarker taxa (**Figure S7A**). *Clostridium_Sensu_Stricto*, *Pseudescherichia*, and *Turicimonas* showed positive correlations with DAI scores, while *Muribaculum*, *Paramuribaculum*, and *Lachnospiracea_incertae_sedis* presented negative correlations. Bacterium including *Limosilactobacillus*, *Lactobacillus*, *Turicibacter*, and *Romboutsia* had highest abundance in intermediate level of DAI scores (**Figure S7C**). These models provided a better understanding of microbial dynamics and corresponded with the result of differential analysis.

### The interactions and networks in gut microbiota

**Figure 4:**
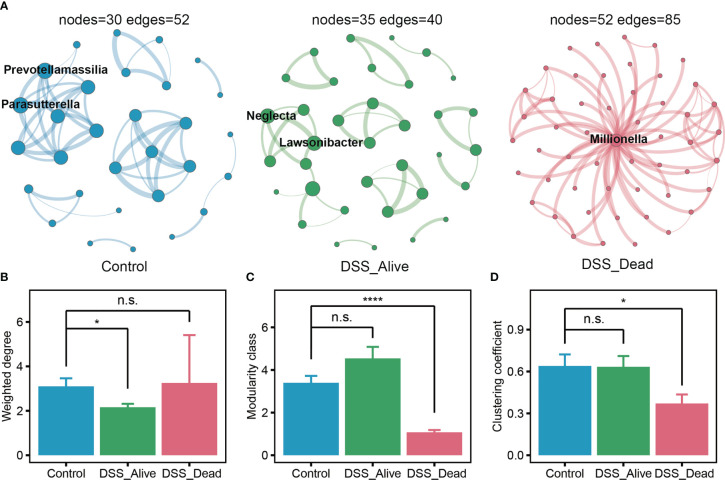
Co-occurrence network of the three group. **(A)** Microbial networks of Control group, DSS_Alive group, and DSS_Dead group. The size of the nodes was proportional to the value of the degree. The thickness of the edges was proportional to the degree of correlation. The properties of network about weighted degree, modularity and clustering coefficient present in **(B–D)**. n.s. means no significant difference, * means *P* < 0.05, **** means *P* < 0.0001.

Further, to reveal the interactions of microorganisms in the colorectum, we performed co-occurrence analysis of genus-level microbial network. All analysis was performed under the same parameters. Suggested by the results, the Control group had 30 nodes and 52 edges, the DSS_Alive group had 35 nodes and 40 edges, and the DSS_Dead group had 52 nodes and 85 edges (**Figure 4A**). And we also noticed that the DSS_Alive group had the highest number of modularity class, but its average number of edges per node (1.1429) was lower than that of the Control group (1.7333) and the DSS_Dead group (1.6346) (**Figures 4B, C**). *Prevotellamassilia* and *Parasutterella* were the hub genus of the Control group, and *Lawsonibacter*, *Oscillibacter*, and *Neglecta* were the hub genus of the DSS_Alive group. They might play a core role in the relevant microbial network. The degree of those hub genus was not very significant (**Table S2**). In contrast, *Millionella* was the hub genus of the DSS_Dead group, which had the highest degree (51) and a large gap compared with other microorganisms. It suggested that *Millionella* was essential for maintaining the stability of the microbial network of the DSS_Dead group, which made the network vulnerable. We also found that interactions between *Neglecta*, *Mediterraneibacter*, and *Paludicola* were simultaneously presented in the DSS treatment group and absent in the Control group, suggesting a correlation between the treatment of DSS and the interactions between these three bacteria. We also analyzed the properties of the microbial network. The results showed that treatment of DSS significantly reduced the degree of the microbial network, lowered its complexity, and decreased its modularity (**Figures 4B–D**). In conclusion, the treatment of DSS led to a decrease in the stability and cohesiveness of the microbial network and made microbial composition simpler.

### Predicted functions of microbial communities

**Figure 5:**
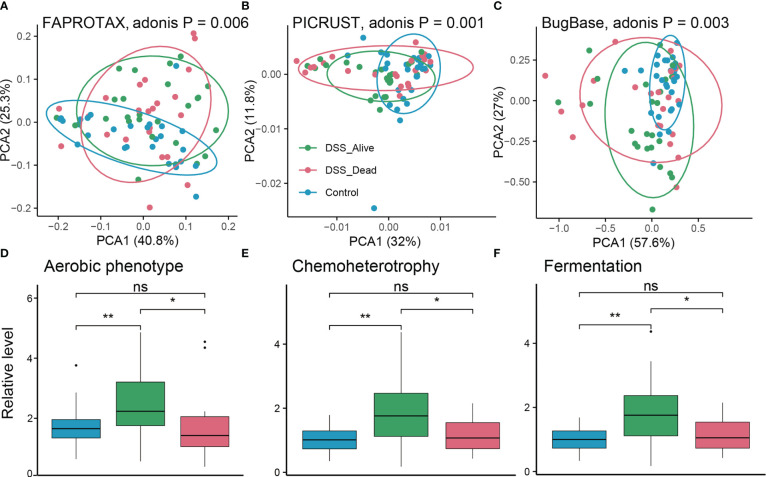
Predicted functions of the three group. PCA plot of predicted functions based on FARPROTAX **(A)**, PICRUST **(B)**, and BugBase **(C)**. P-values were calculated using permutational multivariate analysis of variance (Adonis). Boxplot plot of corresponding significant functions among the three group **(D–F)**. Wilcoxon rank-sum test was used to test significance in three groups. n.s. means no significant difference, * means *P* < 0.05, ** means *P* < 0.01.

To further explain and explore the potential functions of microbial communities, functional predictions were conducted based on algorithms including FAPROTAX, PICRUST, and BugBase. There were significant differences among the Control group, the DSS_Alive group, and the DSS_Dead group. Adonis p values based on functional matrix of FAPROTAX, PICRUST, and BugBase were 0.006, 0.001, and 0.003, correspondingly (**Figures 5A–C**). We further specified differential predicted phenotypes or pathways between the DSS_Alive group and the DSS_Dead group. Three functions were considered significant including aerobic phenotype, chemoheterotrophy, and fermentation. Relative levels of the three function in the DSS_Alive group were significantly higher than those in the Control group and the DSS_Dead group (**Figures 5D–F**). These results revealed significant differences in predicted functions of microbial communities, and promoted understanding of surviving mechanisms in AOM-DSS mice.

## Discussion

As one of the most common methods to study CRC, the success rate of AOM-DSS model in mice has been a long-standing problem (Andrei et al., 2022). In our study, we found that the physiological signs of mice were extremely different among groups during CRC modeling. As the experimental time progressed, the relevant indicators changed. We observed that DSS_Alive and DSS_Dead groups had decreased body weight and their DAI scores were elevated compared to the Control group. They were consistent with previous studies, and these results indicated that the mice were successfully induced to status of inflammatory bowel disease under the pressure of AOM and first round DSS (Chartier et al., 2018; Chartier et al., 2020b; Chartier et al., 2020a). However, mice in DSS_Dead group showed a more obvious weight loss and higher DAI score, which indicated that more severe colitis was the main reason for the death and failure of model construction. It was intriguing to distinguish differences of gut microbial structures based on physiological results of these AOM-DSS treated mice.

The microbial community is generally considered an important biological factor in intestinal diseases (Cheng et al., 2020). Previous studies had shown that CRC patients underwent significant changes in the structure of the gut microbiome. Alterations in microbial composition modulated local responses and produced toxin genes, thus playing a regulatory role in tumor development (Dejea et al., 2018; Garrett, 2019; Janney et al., 2020). Our resulted suggested that in the early stage of AOM-DSS model, gut microbiota were inclined to appear phenotype of colitis rather than colorectal cancer. In this study, we found that the correlation between the microbial structure and experimental time was different in the DSS_Alive and DSS_Dead groups. The DSS_Alive group gradually reached the recovery phase. Concurrently, the mice weight recovered and the microbiota composition became similar. In contrast, the DSS_Dead group had rapid changes in intestinal microbial structure due to breakout of colitis. These were consistent with previous findings in acute and chronic colitis (Xu et al., 2021; Zhou et al., 2021). Mice treated with AOM-DSS would fail to convert microbial composition from acute to chronic colitis if severe colitis was induced. Significant microbes might have the potential to control colitis levels and decide fate of AOM-DSS treated mice.

In the early stage of AOM-DSS model, we found that there were significant differences in microbial composition between the DSS_Alive and DSS_Dead groups. By differential analysis, we observed that the relative abundance of *Pseudescherichia* in the DSS_Dead group was significantly increased to over 50% Studies have found that *Pseudocherichia vulneris* had close relatives with the pathogenic bacterium *Escherichia vulneris*, suggesting that *Pseudocherichia* might have a potential pathogenic role and deserved further investigation in the future (Alnajar and Gupta, 2017; Fan et al., 2021). We also noted that the relative abundance of *Turicimonas* and *Clostridium_XVIII* in the DSS_Dead group significantly increased at the end of induction phase. There were few studies related to *Turicimonas*, while *Clostridium_XVIII* was shown to produce exotoxins and promote inflammation with proinflammatory potential (Matsuda et al., 2000; Stiles et al., 2014; Woting et al., 2014). These genera, especially *Pseudocherichia*, might play an significant role in causing severe colitis and might be one of the main reasons for the death of AOM-DSS treated mice at the end of induction phase.

Concurrently, the relative abundance of *Ruthenibacterium* and *Akkermansia* were increased in mice surviving from AOM-DSS treatment. They preempted the niche of pathogenic bacteria at the end of induction phase, which could reduce damage of pathogenic bacteria and the mortality of model mice. There were few researches on *Ruthenibacterium*. *Akkermansia*, with relative abundance over 10% in the DSS_Alive group, is considered as a genus of gut beneficial bacteria. It can promote an anti-inflammatory and antioxidant status in the gut by increasing the production of short chain fatty acids (SCFAs) (Zhai et al., 2019). The significant increase in the relative abundance of the two genera at the end of induction phase might be of great significance for alleviating colorectal damage and preventing death of model mice.

In the convalescent phase, the relative abundance of *Ligilactobacillus*, *Limosilactobacillus*, and *Lactobacillus* returned to the normal level while *Pseudocherichia* was at a relatively low level. The beneficial effects of *Lactobacillus* have been widely reported, and many clinical studies have shown its ability to reduce chronic inflammation associated with cancer development (Salva et al., 2014; Shin et al., 2016; Zhuo et al., 2019). Studies have proven that probiotic bacteria such as *Lactobacillus* were able to inhibit deterioration of CRC by secreting SCFAs, suppressing inflammation and angiogenesis, and enhancing the function of the intestinal barrier (Chattopadhyay et al., 2021). *Ligilactobacillus* is another common probiotic. It could increase microbiota abundance of colorectum and alleviate symptoms of IBD by reducing serum inflammatory cytokine, declining bacterial translocation levels, and achieving protective effects on the barriers of colorectum (Shi et al., 2017; Alard et al., 2018). Therefore, the increase of these probiotics suggested that they gradually exerted protective effects on the colorectum and played an important role in the alleviation of the disease in model mice. It suggested a critical role of the three probiotics for the transition from acute to chronic colitis.

In general, the relative abundances of *Pseudocherichia*, *Turicimonas*, and *Clostridium_XVIII* were significantly elevated in the DSS_Dead group and might occupy niches of the other probiotics. In the convalescence, the relative abundance of beneficial bacteria returned to the normal level or even significantly increased, while the relative abundance of pathogenic bacteria was greatly reduced. This trend reduced the damage effect of pathogenic bacteria and promoted the beneficial bacteria to exert protective effects on the colorectum. Successful transition in the early stage of AOM-DSS model relied on sufficient niches of gut beneficial bacteria in induction phase and convalescence.

From the perspective of network properties, we found that the Control group had the highest average number of edges per node and a lower number of modularity classes compared to DSS_Alive and DSS_Dead groups. These meant that the co-occurrence network of Control group had the higher connectivity and stronger cohesion, which made it more stable. Compared to groups of Control and DSS_Alive, the DSS_Dead group had the most nodes and edges. However, *Millionella* was the only hub genus maintaining the entire network, which made the network vulnerable. There was very little known about *Millionella*. It was first isolated from human right colon in 2017, which has only been reported as a bacterium capable of promoting obesity and associated with liver injury and insulin resistance (Mailhe et al., 2017; Zhang et al., 2020; Ma et al., 2022). It might also function as a potential diagnostic biomarker for dysbiosis of the intestinal flora ecology (Zhang et al., 2020). More studies were needed to elucidate the properties and functions of *Millionella* in the future. The simplification and instability of the gut flora network might also be one of the reasons for the failure of the AOM-DSS model in the early stage of construction.

Potential functions were increased in the DSS_Alive group including aerobic phenotype, chemoheterotrophy, and fermentation. The significant function simultaneously modulated both microbial and host pathways, and changed rapidly in human infants (Li et al., 2022). SCFAs, productions of microbial fermentation, not only regulate community stability of gut microbiota, but underlies adaptive homeostasis and colonic health as well (Wong et al., 2006; Smith et al., 2013). Increased fermentation in emergency might play a vital function in promoting colonic homeostasis and health in AOM-DSS treated mice. These results corresponded with significant probiotics in the DSS_Alive group, and helped better understand their potential mechanisms.

In conclusion, our study showed that microorganisms played an important role in the construction of CRC model. We focused on the early stage of AOM-DSS model, conducted ecological and dynamic analysis, and provided a better understanding for the shift of gut microbiota in AOM-DSS treated mice. Some microbes might perform a vital function in the successful construction of AOM-DSS model.

## Data availability statement

The original contributions presented in the study are included in the article/**Supplementary Material**. Further inquiries can be directed to the corresponding authors. The 16S rRNA gene data reported in this paper have been deposited in the Sequence Read Archive (https://www.ncbi.nlm.nih.gov/sra), under accession number PRJNA940365 (https://www.ncbi.nlm.nih.gov/bioproject/PRJNA940365/).

## Ethics statement

The animal study was reviewed and approved by the Laboratory Animal Ethics Committee of Xiangya Hospital, Central South University.

## Author contributions

Study design: CL and JH. Data collection: RS, HC, and SY. Data analysis: RS, HC. Manuscript writing: RS. Technical guidance: ZY. All authors contributed to the article and approved the submitted version.
