# Supplementary material for: Ecological and dynamic analysis of gut microbiota in the early stage of azomethane-dextran sodium sulfate model in mice
Source: Front Cell Infect Microbiol. 2023 Apr 20;13:1178714. doi: 10.3389/fcimb.2023.1178714 (PMC10157258; doi:10.3389/fcimb.2023.1178714)
Supplement: Supplementary file 1 [file DataSheet_1.pdf]

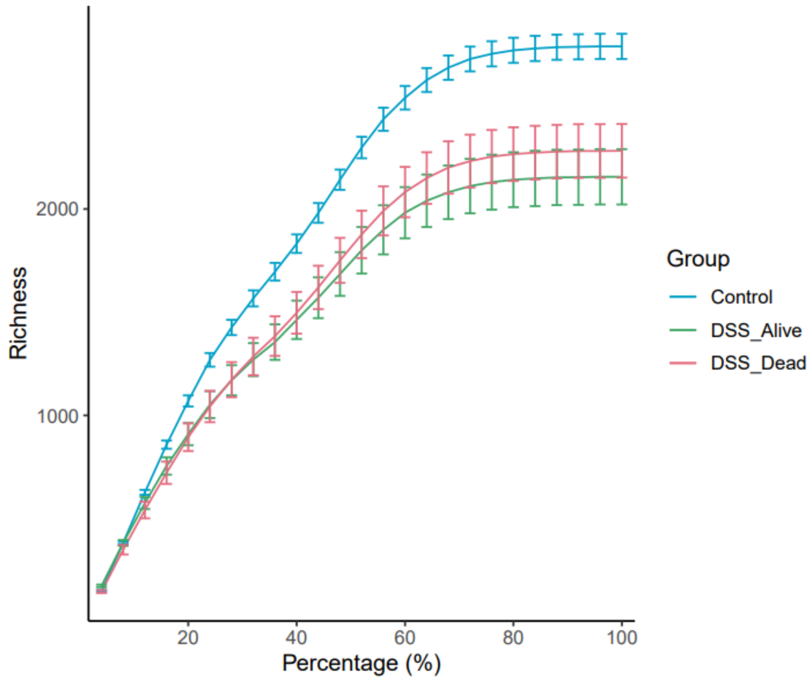

Figure.S1 Rarefaction curves of the three group.

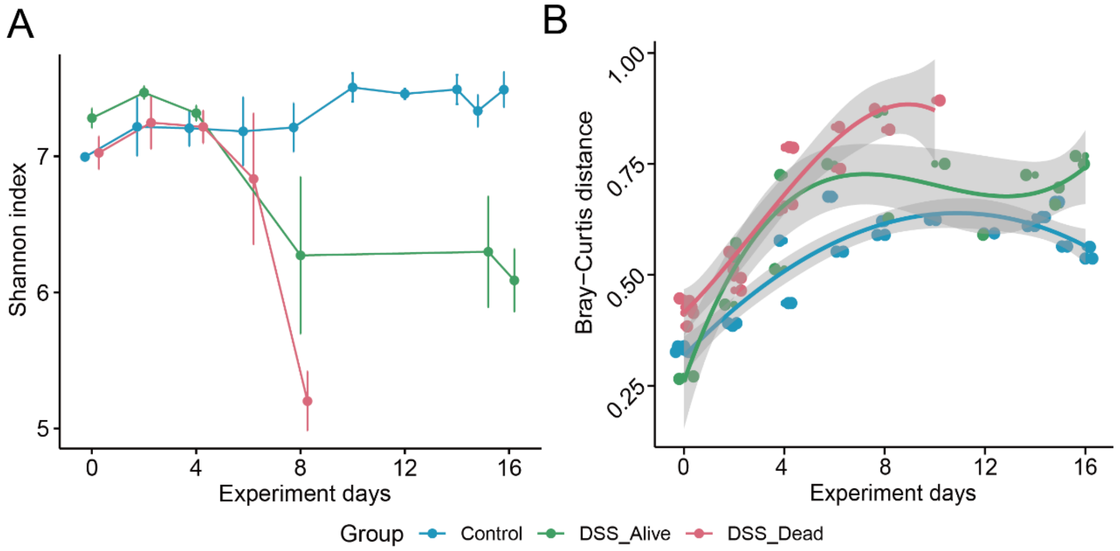

Figure.S2 The alpha and beta diversity of the three group over experiment time. (A) Changes of Shannon index over time for three groups. (B) The change of Bray-Curtis distances over experiment time.

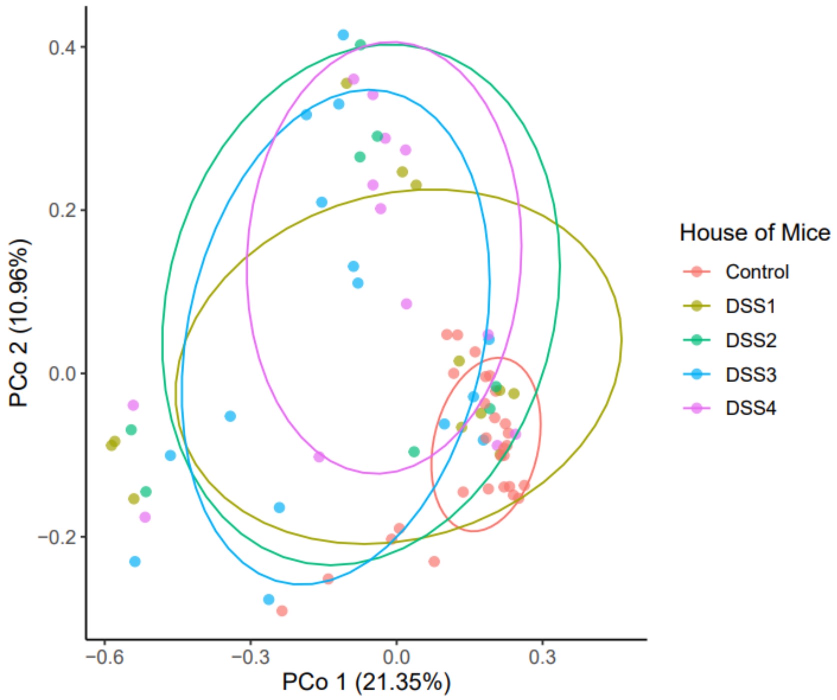

Figure.S3 Beta diversity of different cages analyzed by PCoA.

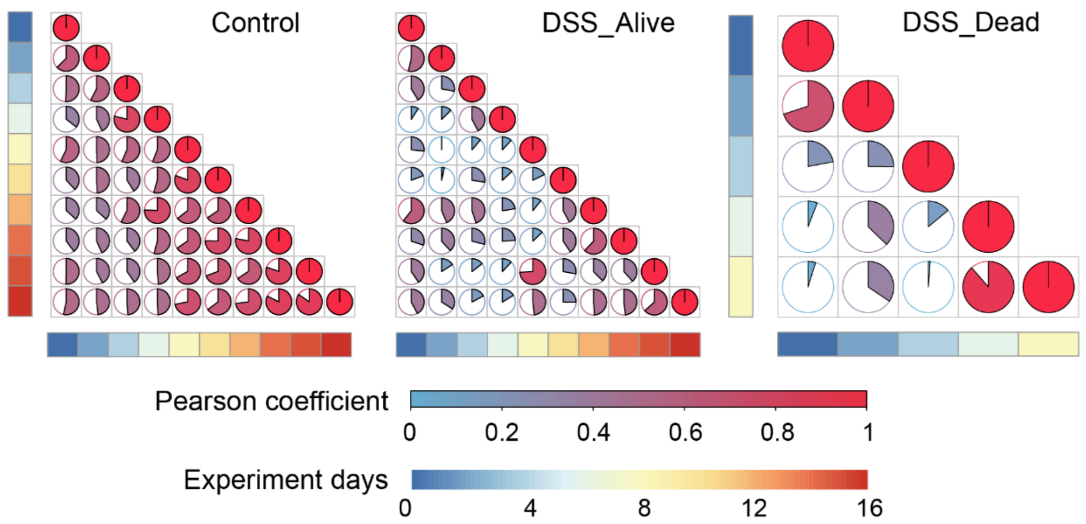

Figure.S4 The analysis of beta diversity within the three group. Pearson correlation analysis showing the correlation between experimental time and changes in microbial community structure for each group in the Control, DSS_Alive and DSS_Dead. The area and color of each pie chart means the Pearson coefficient of two corresponding samples.

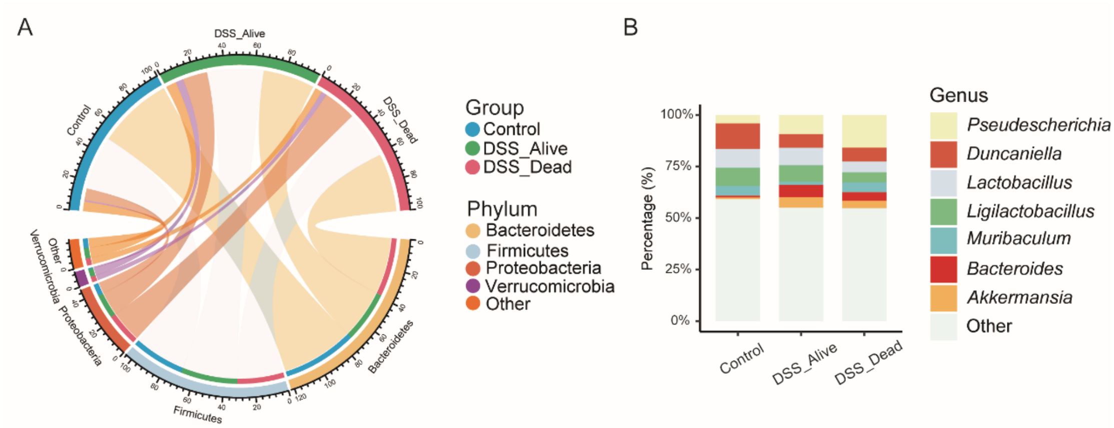

Figure.S5 The analysis of microbial composition at different taxonomic levels. The figures present the microbial composition at the levels of phylum (A) and genus (B).

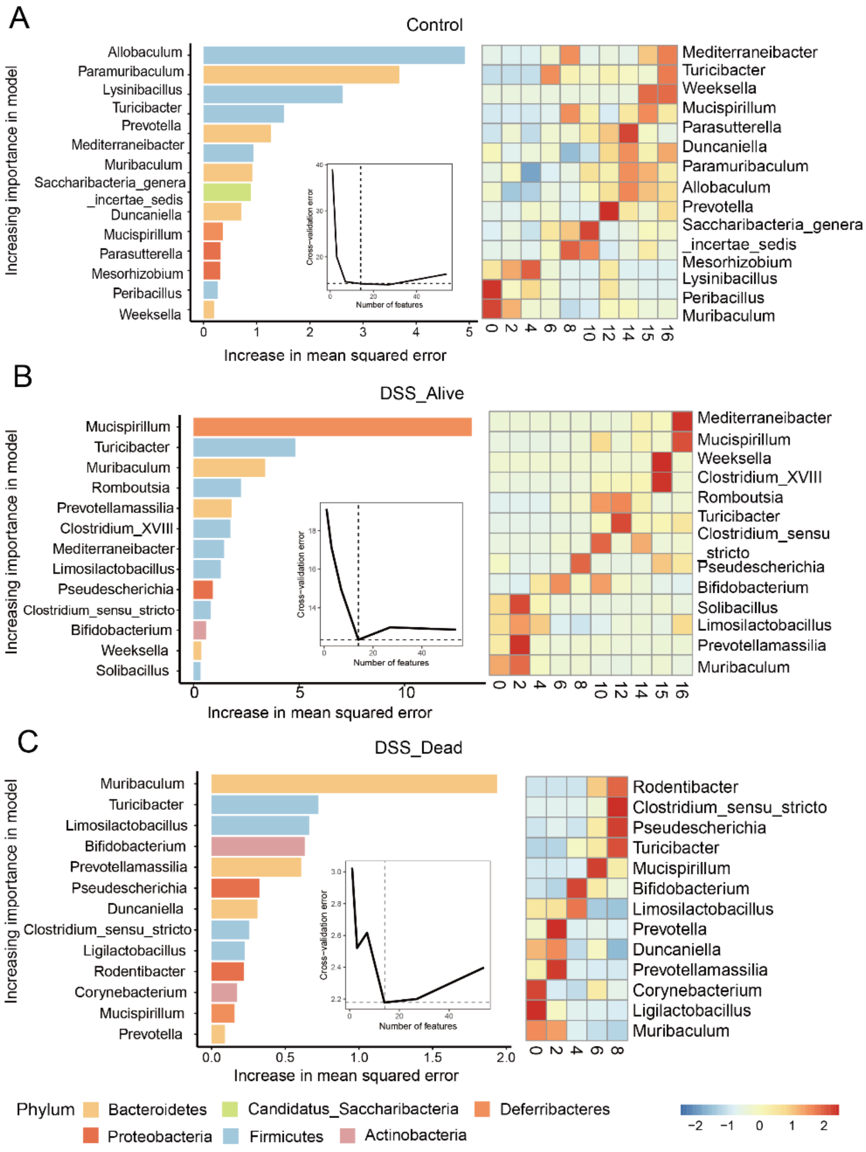

Figure.S6 Biomarkers of progression over time among the three group. The importance of biomarkers Control group (A), DSS Alive group (B), and DSS Dead group (C) through machine learning. The heat maps were used to show changes in key genera over the time of the experiment. Heat map color bars represent correlations.

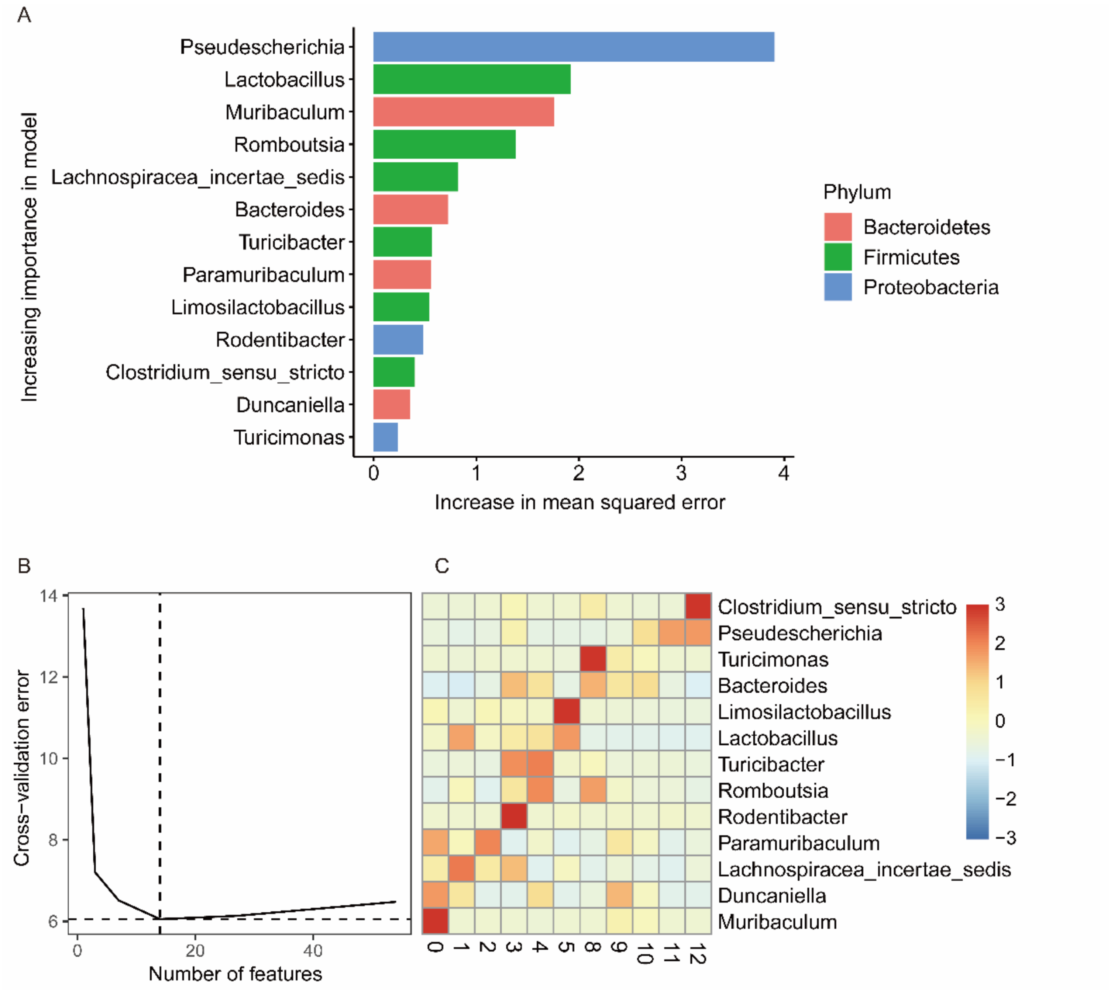

Figure.S7 The relationship between DAI scores and biomarkers. (A) The top 13 biomarkers based on DAI scores by applying Random Forests regression. (B) The Cross-validation of Random Forests regression. (C) Heatmap of the top 13 biomarkers and DAI scores. Heatmap color bars represent correlations.

Table.S1 Adonis test of microbial composition in different groups.

	Variable1	Variable2	Pvalue of adonis
**Grouped by housing**	Control	DSS1	0.0003
	Control	DSS2	0.0001
	Control	DSS3	0.0001
	Control	DSS4	0.0001
	DSS1	DSS2	0.5221
	DSS1	DSS3	0.3300
	DSS1	DSS4	0.2071
	DSS2	DSS4	0.1949
	DSS2	DSS3	0.2835
	DSS3	DSS4	0.4587
**Grouped by treatment and outcome**	DSS_Alive	DSS_Dead	0.0496
	DSS_Alive	Control	0.0001
	DSS_Dead	Control	0.0001

Table.S2 Degrees of hub genus in different groups.

Genus	Group	Degree
Prevotellamassilia	Control	7
Parasutterella	Control	7
Lawsonibacter	DSS_Alive	4
Neglecta	DSS_Alive	4
Oscillibacter	DSS_Alive	4
Millionella	DSS_Dead	51

Table.S3 Metadata of the 16s rRNA gene sequencing.

Mice	Group	Day
T036	Control	0
S045	Control	0
T016	Control	0
T036	Control	2
S045	Control	2
T016	Control	2
T036	Control	4
S045	Control	4
T016	Control	4
T036	Control	6
S045	Control	6
T016	Control	6
T036	Control	8
S045	Control	8
T016	Control	8
S045	Control	10
T016	Control	10
S045	Control	12
T036	Control	12
S045	Control	14
T016	Control	14
T036	Control	14
S045	Control	15
T016	Control	15
T036	Control	15
S045	Control	16
T016	Control	16
T036	Control	16
T035	DSS_Alive	0
S034	DSS_Alive	0
S097	DSS_Alive	0
S034	DSS_Alive	2
S035	DSS_Alive	2
S097	DSS_Alive	2
T035	DSS_Alive	2
S034	DSS_Alive	4
S097	DSS_Alive	4
T035	DSS_Alive	4
S035	DSS_Alive	6
S034	DSS_Alive	8
S035	DSS_Alive	8
T035	DSS_Alive	8

S034	DSS_Alive	10
S097	DSS_Alive	12
S034	DSS_Alive	14
S097	DSS_Alive	14
S034	DSS_Alive	15
S097	DSS_Alive	15
T035	DSS_Alive	15
S034	DSS_Alive	16
S035	DSS_Alive	16
S097	DSS_Alive	16
T035	DSS_Alive	16
S038	DSS_Dead	0
S042	DSS_Dead	0
S043	DSS_Dead	0
T004	DSS_Dead	0
T012	DSS_Dead	0
T018	DSS_Dead	0
S038	DSS_Dead	2
S042	DSS_Dead	2
T004	DSS_Dead	2
T012	DSS_Dead	2
T018	DSS_Dead	2
S042	DSS_Dead	4
S043	DSS_Dead	4
T004	DSS_Dead	4
T012	DSS_Dead	4
T018	DSS_Dead	4
S038	DSS_Dead	6
S043	DSS_Dead	6
T018	DSS_Dead	6
S042	DSS_Dead	8
S043	DSS_Dead	8
T012	DSS_Dead	8
